# A belief-based model for characterizing the spread of awareness and its impacts on individuals' vaccination decisions

**DOI:** 10.1098/rsif.2014.0013

**Published:** 2014-05-06

**Authors:** Shang Xia, Jiming Liu

**Affiliations:** 1 National Institute of Parasitic Diseases, Chinese Center for Disease Control and Prevention, Shanghai, People's Republic of China; 2 Department of Computer Science, Hong Kong Baptist University, Hong Kong

**Keywords:** vaccination decision, awareness, belief model

## Abstract

During an epidemic, individuals' decisions on whether or not to take vaccine may affect the dynamics of disease spread and, therefore, the effectiveness of disease control. Empirical studies have shown that such decisions can be subjected to individuals' awareness about disease and vaccine, such as their perceived disease severity and vaccine safety. The aim of this paper is to gain a better understanding of individuals' vaccination behaviour by modelling the spread of awareness in a group of socially connected individuals and examining the associated impacts on their vaccination decision-making. In our model, we examine whether or not individuals will get vaccinated as well as when they would. In doing so, we consider three possible decisions from an individual, i.e. to accept, to reject, and yet to decide, and further associate them with a set of belief values. Next, we extend the Dempster–Shafer theory to characterize individuals' belief value updates and their decision-making, having incorporated the awareness obtained from their connected neighbours. Furthermore, we examine two factors that will affect individuals' vaccination decisions: (i) reporting rates of disease- and vaccine-related events, and (ii) fading coefficient of awareness spread. By doing so, we can assess the impacts of awareness spread by evaluating the vaccination dynamics in terms of the number of vaccinated individuals. The results have demonstrated that the former influences the ratio of vaccinated individuals, whereas the latter affects the time when individuals decide to take vaccine.

## 1. Introduction

In controlling the spread of infectious diseases, the effectiveness of a vaccination programme depends on the ratio of the vaccinated host population [1–4]. For example, vaccination can prevent disease transmissions when the coverage of a host population is above the critical level of the herd immunity threshold [5,6]. In this context, individuals' vaccination decisions on whether or not to take vaccine can play an important role in achieving adequate and sustained vaccination coverage [7,8].

Previous studies on individuals' vaccination decision-making have typically focused on several determinants with respect to individuals' perceived benefits and risks of vaccination, including vaccine-induced immunization [9–11], the possible adverse events following immunization (AEFI) [12,13] as well as social and financial costs associated with disease infection and vaccination, such as the direct costs of vaccination, the expenses for the treatment of disease infection, and the indirect costs in the form of absence from workplaces or schools [14–16]. In this regard, game-theoretical analysis has been widely used to describe individuals' vaccination decisions by examining their personal optimized pay-offs based on the perceived risks and benefits of vaccination [17–19]. Moreover, some studies have looked into the social and psychological aspects of decision-making (e.g. social learning process [20] and imitation behaviour [21–24]). While others have considered the issues of incomplete information by adding either the potential discrepancy between individuals' perceptions and real situations (e.g. the perceived disease prevalence and the adverse effects of vaccine [25,26]) or different sources of information (e.g. previous disease prevalence or vaccination programmes [27–29]).

These earlier decision-making studies have addressed the problem of whether or not individuals will get vaccinated by taking into account their prior knowledge about the disease, the vaccine and the associated costs. While during a vaccination programme, the time when individuals will make their vaccination decisions is still a concern. Furthermore, the above-mentioned prior knowledge may exist only for the routine vaccination programmes against seasonal infectious diseases (e.g. measles and chickenpox [30]). As for a newly developed vaccine against an emerging infectious disease (e.g. vaccine against the 2009 H1N1 influenza [31]), there is always a lack of such prior knowledge. Empirical studies have shown that individuals' vaccination decisions can be subjected to their perceptions about disease and vaccine rather than to the actual situations, which are continuously affected by the social environment with which they interact [32]. Specifically, individuals who are aware of severe disease infections will tend to seek protection from vaccination. For instance, the impacts of a measles epidemic were observed to increase the uptake of measles vaccines [33]. On the other hand, when realizing the potential risks of vaccine, e.g. vaccine-related AEFI, individuals will reduce their willingness to vaccinate themselves, such as in the case of the MMR vaccine (i.e. vaccine against measles, mumps and rubella) scare in the UK in the 1990s [34,35]. In this regard, individuals' awareness about severe disease infections and vaccine-related AEFI will affect their perceptions about disease severity and vaccine safety, and hence, influence their vaccination decisions. In addition, an individual changing his/her vaccination decision does not require direct self-involvement in the reported events, while being informed through others. That is to say, individuals' perceptions can be prompted through the spread of awareness in a host population [36,37], which can potentially alter individuals' vaccination decisions and, hence, affect the effectiveness of vaccination programmes.

In recent years, the rapid emergence of online social media, such as Facebook [38], Twitter [39] and YouTube [40,41], have provided new ways for the spread of public-health-related information [42]. As for vaccination, the online social communities would debate on the efficacy of vaccines [43]; the vaccine-related AEFI would be reported and shared on the Internet [44], and opinions either for or against vaccination would be transmitted from person to person [37]. In this situation, the spread of awareness about disease and vaccine could immediately affect individuals' responses. Therefore, the dynamics of individuals' vaccination decisions will be tightly coupled with that of disease transmissions. The interplay between these two dynamical processes could have a significant consequence on the resulting vaccination coverage for infectious disease control.

**Figure 1.**
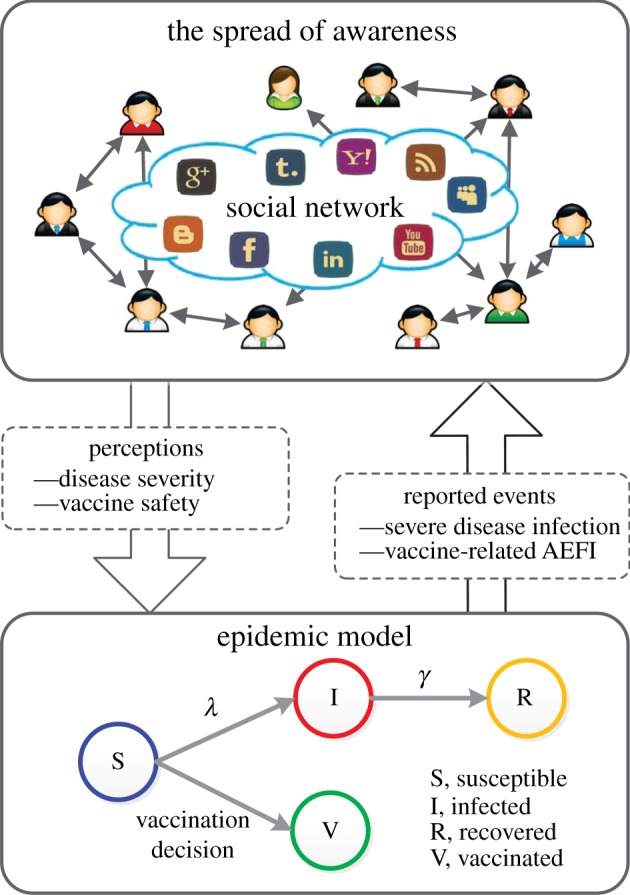
A schematic of the impacts of the spread of awareness on individuals' vaccination decision-making. We consider a group of individuals whose vaccination decisions (i.e. whether or not to take vaccine) depend on their perceptions about disease severity and vaccine safety. We use a social network to characterize the structure of individuals' interactions. The awareness about severe disease infections and vaccine-related AEFI can spread from person to person through their interaction relationships, which will substantially affect their perceived disease severity and vaccine safety and, thus, change their vaccination decisions accordingly. (Online version in colour.)

In this study, we are interested in studying individuals' vaccination decisions as affected by the spread of awareness about disease and vaccine-related events during an influenza-like epidemic. As illustrated in figure 1, we consider a group of individuals that can decide whether or not to take vaccine based on their perceived disease severity and vaccine safety. Specifically, individuals can interact with each other through their social relationships (e.g. friendships on the Facebook and follower relationships on the Twitter). In such a structured host population (i.e. represented by a social network), the awareness about disease and vaccine can spread from person to person and will substantially affect individuals' perceptions about disease severity and vaccine safety. On the one hand, the reported cases of severe disease infections will enhance individuals' perceived disease severity and, hence, increase their tendency to vaccination. On the other hand, the reported events of vaccine-related AEFI will weaken the public confidence on vaccine safety, which will lead to the declined acceptance of vaccination.

In this situation, we develop a novel modelling framework for characterizing individuals' vaccination decisions, in which we suppose that an individual will voluntarily decide to accept or reject vaccine based on his/her beliefs on whether or not vaccination is acceptable. In order to examine the time when individuals will make their vaccination decisions, we further assume that if the individual does not have enough confidence for or against vaccination, he/she will not make any firm decision but wait and see the future development. The situation that an individual makes no firm decision may be considered as a state of ‘yet to decide’ owing to uncertainty. In this regard, we introduce three belief variables in the form of *yes*, *no* and *no decision* to characterize the possible decision responses from an individual that he/she will accept or reject vaccine, or has not yet decided, respectively. Owing to the spread of awareness, the individual will update his/her beliefs about vaccination by collecting information from his/her social neighbours, which may either reinforce his/her own perceptions, or bring about conflicting perceptions.

In order to characterize individuals' belief value updates based on the obtained awareness and their subsequent vaccination decision-making in the presence of uncertainty, we develop a new *belief–decision* model by extending the framework of the Dempster–Shafer theory (DST) [45]. DST, also known as theory of beliefs, was originally proposed as a generalization of the Bayesian theory of subjective probability to characterize how individuals update their beliefs by combining new pieces of evidence from multiple sources in the presence of uncertainty [46]. In our proposed DST-based belief–decision model, individuals can update their beliefs (i.e. with respect to perceived disease severity and vaccine safety) by combining the collected new evidence from social neighbours (i.e. the obtained awareness about disease and vaccine). Furthermore, we extend the conventional DST framework by incorporating the effect of awareness spreading, in which a reported event about disease or vaccine (i.e. a piece of new evidence) will ripple through individuals' social network. In this regard, individuals' vaccination decision-making is modelled as a process being affected by the spread of awareness about disease and vaccine as well as the subsequent updates of individuals' beliefs (i.e. the belief values of both yes and no).

We parametrize our proposed model with an influenza-like disease as well as a social network from a real-world online community. By carrying out a series of simulations on voluntary vaccination and infectious disease transmissions, we evaluate the impacts of the spread of awareness on individuals' vaccination decisions as well as its consequence on disease transmission dynamics with respect to the following two impact factors: (i) reporting rates of disease- and vaccine-related events, which denote the probabilities for an infected or vaccinated individual to be reported as a case of severe disease infections or vaccine-related AEFI and (ii) fading coefficient of awareness spread, which describes the effect of certainty decay when the awareness spreads from one person to another.

## 2. Models

We consider a voluntary vaccination programme for preventing the outbreak of an emerging infectious disease, e.g. 2009 H1N1 influenza, in which individuals can decide whether or not to take vaccine based on their awareness about disease severity and vaccine safety. It is assumed that individuals do not possess any prior knowledge about disease and vaccine, whereas they can receive information about disease- and vaccine-related events (i.e. the reported severe disease infections and vaccine-related AEFI). In this situation, the reported event about either disease or vaccine will trigger the spread of awareness among the host individuals, rippling through their interaction relationships, which will, in turn, affect their vaccination decisions.

For such a situation, we construct a new individual-based *belief–decision* model to characterize vaccination decision-making. At the same time, we use an epidemic model to describe the dynamics of disease transmission as a result of individuals' voluntary vaccination. Based on our constructed model, we aim to investigate the impacts of the spread of awareness on the changes of individuals' vaccination decisions with respect to an emerging infectious disease. The parameters as used in the proposed model are summarized in table 1.

**Table 1.** Parameters in the belief–decision model.

symbol	description
*m*(Yes)	belief value of vaccination
*m*(No)	belief value of non-vaccination
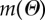	belief value of no decision (uncertainty)
*m_i_*	set of belief values
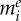	obtained awareness about disease and vaccine
*ρ*	fading coefficient of awareness spread
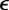	reporting rate of severe disease infections
*κ*	reporting rate of vaccine-related AEFI
*S*(*t*)	number of susceptible individuals
*I*(*t*)	number of infectious individuals
*R*(*t*)	number of recovered/immunized individuals
*N*	total number of host individuals
*λ*(*t*)	probability of disease infection
*β*	disease transmission rate
*γ*	infection recovery rate
*R*_0_	basic reproduction number

### 2.1. The belief–decision model

As described above, we have considered three possible vaccination-related decisions, i.e. to accept, to reject and yet to decide. For each individual, we first represent his/her willingness to accept or reject vaccine by using a set of belief variables. In order to characterize the state of ‘yet to decide’, we introduce the notion of decision-making with uncertainty based on the DST [45]. DST can be viewed as a generalization of the Bayesian theory of probability. Unlike the Bayesian theory, DST explicitly allows for an undecided state with respect to the presently available knowledge. We suppose that the problem of whether or not to take vaccine is a binary problem, which is represented as 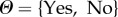 called the frame of discernment for the vaccination decisions (i.e. a universal set). Individuals' possible vaccination decision responses can be modelled as the subsets of *Θ*, i.e. belonging to a power set, 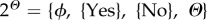. Next, we use a function *m*(·) to assign a belief mass (i.e. probability) to each element of the power set 2^*Θ*^, which is called the basic probability assignment (BPA). The mass *m*(A) 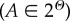 denotes the proportion of support for the particular subset *A* based on the currently available evidence or knowledge. The BPA has the following two properties: (i) the mass of empty set *ϕ* is zero, and (ii) the masses of the power set add up to one2.1
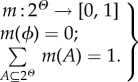
Accordingly, the belief functions for an individual's vaccination decision responses can be expressed as follows2.2
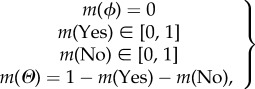
where *m*(Yes) describes an individual's belief that he/she should get vaccinated for preventing disease infection, *m*(No) represents the belief that he/she should reject vaccination having considered the potential risk of vaccine-related AEFI, and *m*(*Θ*) denotes the belief that he/she is yet to decide whether or not to get vaccinated (i.e. owing to the uncertainty about disease and vaccine). Based on the above formulation, an individual will decide to take vaccine with the probability of *m*(Yes), reject vaccine with the probability of *m*(No), and have no firm decision with the probability of *m*(*Θ*). In addition, we assume individuals with the decision of vaccination will get vaccinated directly and, therefore, will either be successfully immunized or suffer from vaccine-related AEFI. Those with no decision will revise their decisions in the next time step. We then use the DST to characterize individuals' decision-making with reference to information from multiple sources.

### 2.2. The spread of awareness

During the spread of an emerging infectious disease and the implementation of a vaccination programme, individuals' perceptions about disease severity and vaccine safety will be affected by the obtained awareness from their socially connected neighbours. For instance, a case of severe disease infection can be naturally regarded as the evidence that an individual should get vaccinated. On the other hand, the events of vaccine-related AEFI can be viewed as the evidence that vaccination may be rejected. Here, we use a belief value *m^e^* to denote a piece of evidence that is triggered from a newly reported disease- or vaccine-related event. In order to characterize the spread of awareness in a socially connected host population, we consider two rules of information dynamics: (i) information transmission that awareness will spread between two connected individuals, and (ii) awareness fading that the certainty of a piece of new evidence will be gradually lost as of each transmission [47].

We suppose that a group of individuals are socially interconnected through their social network, denoted by 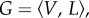 where 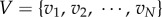 is the set of nodes (i.e. individuals), and 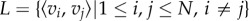 is the set of links (i.e. social interaction relationships). *N* is the total number of individuals. During an epidemic, each reported event will be treated as a piece of triggering evidence with a belief value of 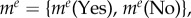 where 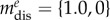 and 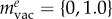 for a reported case of severe disease infection- and vaccine-related AEFI, respectively. Individuals can detect the emerged new evidence by interacting with their social neighbours, update their belief values accordingly, and can further talk about it to others through their social networks. Additionally, the certainty about a piece of evidence will decay as it is transmitted from person to person, which is referred to as awareness fading. Here, we introduce a fading coefficient, *ρ*, to indicate how fast the decay will be when transmitting a piece of evidence between two individuals. A larger value of *ρ* corresponds to a faster decay (i.e. faster certainty fading). Therefore, the evidence that is transmitted (i.e. the spread of awareness) from individual *j* to his/her socially connected neighbour *i* can be computed as follows2.3
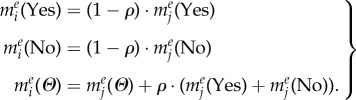


In the course of disease transmissions and vaccination implementation, newly reported events will constitute new sources of evidence at different time steps. The spread of awareness about these events will cause an individual to continuously update his/her perceptions about disease and vaccine, and thus make his/her vaccination decision. Based on the obtained awareness, the individual will update his/her belief values (i.e. denoted by 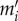) by combining the present belief values (i.e. denoted by *m_i_*) with the newly received evidence 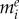. This can be expressed in the following form (with 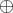 denoting the combination operation):2.4
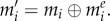


Specifically, based on the assumption that these multiple sources are independent, the belief value update with respect to the extended Dempster rule of combination [48] will be performed as follows2.5
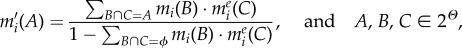
where 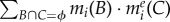 represents the basic belief mass associated with the conflicts of present beliefs and the newly received evidence. In the Dempster combination rule, the denominator, 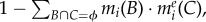 is a normalization factor, which attributes the conflict probability mass to the universal set 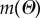. In the extreme case, when an individual with the belief values of {1.0, 0} incorporates the evidence with the belief values of {0, 1.0}, his/her updated belief values will become {0,0}, which means the two conflicting opinions will lead the value of the individual's uncertainty 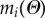 to unity.

**Figure 2.**
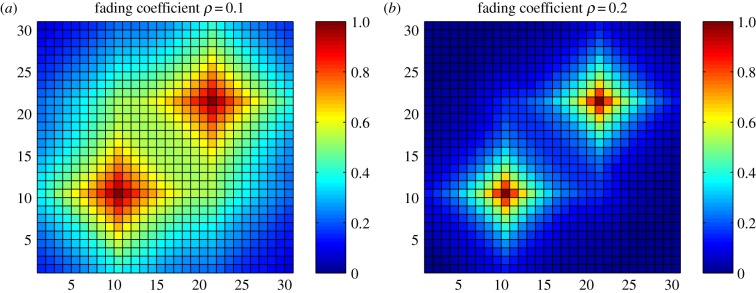
Illustrations of awareness spreading and fading on a synthetic lattice network (i.e. 30 × 30 nodes). Individuals can interact with their socially connected neighbours. The awareness about the two reported events will spread independently in such a structured host population, which will affect individuals' belief values. Here, we use the colour of a cell to denote an individual's belief value in terms of the probability mass (i.e. *m_i_∈*(0, 1)). As for the source of a reported event, the probability mass is set as *m_i_* = 1.0. We use the parameter *ρ* to denote the fading coefficient of awareness spread. A larger value of *ρ* indicates a faster decay of certainty as the awareness spreads from one person to another. We demonstrate the effects of the awareness fading in terms of individuals' belief values (i.e. the colour of each cell) with respect to two considered coefficients: (*a*) *ρ* = 0.1 and (*b*) *ρ* = 0.2.

By doing so, we have developed a belief–decision model for characterizing individuals' vaccination decisions in the presence of uncertainty by using the DST. Furthermore, we have extended the classical DST framework by incorporating the spread of awareness in a structured host population, in which the certainty about a piece of evidence will decay as it is transmitted from person to person. For the sake of illustration, figure 2 shows the results of individuals' belief value updates with respect to the spread of awareness about two independently reported events on a synthetic lattice network.

### 2.3. The epidemic model

We construct an epidemic model to characterize the spread of an emerging infectious disease in a host population, in which the events (e.g. severe disease infections and vaccine-related AEFI) will be reported and, hence, the awareness will spread among them. In doing so, we adopt a standard compartmental model, i.e. susceptible–infectious–recovered (SIR) model, to describe the dynamics of disease infection, in which individuals are grouped into one of three infection-associated, homo-mixed compartments: susceptible (*S*), infectious (*I*) and recovered/immunized (*R*). Therefore, the dynamics of disease transmission, as reflected in the dynamically changing compartments *S*(*t*), *I*(*t*) and *R*(*t*), can be modelled by using the following equations2.6
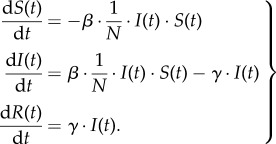


Here, *β* is the disease transmission rate between the susceptible and infectious populations. *γ* represents the recovery rate that is the percentage of infectious individuals who will be recovered per time unit. Based on the definition of basic reproduction number 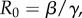 transmission rate can be calculated as 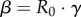. Thus, the probability of being infected for a susceptible individual, denoted by *λ*(*t*), can be computed as follows2.7
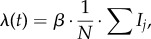
where *I_j_* denotes individual *j* is infected at time *t*, and *N* is the total number of host individuals. Therefore, 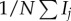 is the ratio of the infectious population. In this situation, we suppose that there is a probability 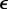 for each newly infected individual to be reported as a case of severe disease infection.

In addition, for the sake of example, we assume that vaccine is available and adequate at the same time as disease onsite (i.e. the first case of disease infection being reported). Only the susceptible individuals can decide whether and when to be vaccinated. Once an individual is vaccinated, it is assumed that he/she will be completely immunized and move from the susceptible compartment to the recovered/immunized compartment. As for a vaccinated individual, there is a probability *κ* for being announced as a case of vaccine-related AEFI.

## 3. Results

### 3.1. Basic scenario

**Figure 3.**
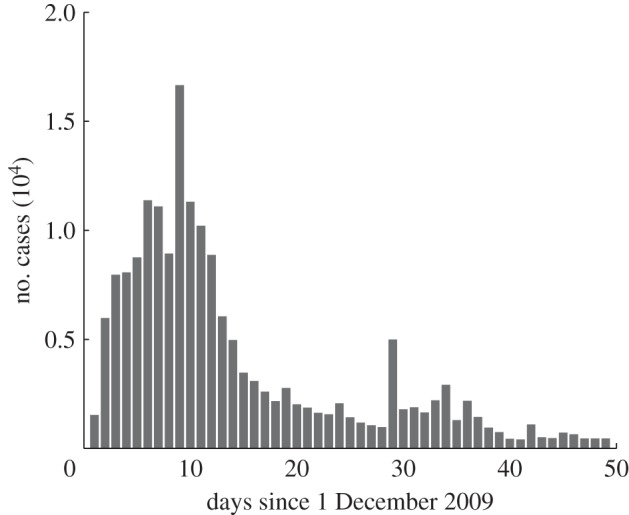
The number of daily-vaccinated individuals during the human swine influenza (HSI) vaccination programme against the 2009 Hong Kong H1N1 influenza epidemic. This programme was launched on 1 December 2009. As of 13 March 2010, more than 180 000 doses of HSI vaccines were administered to persons of various groups [52]. The rate of AEFI was evaluated as 17.8 per 100 000 vaccinated individuals and the reporting rate of vaccine-related AEFI *κ* was estimated as 0.0178%.

For our simulations, we calibrate the parameters in the proposed epidemic model with reference to the scenario of the 2009 Hong Kong H1N1 influenza epidemic, in which basic reproduction number *R*_0_ in the epidemic stage was estimated as *R*_0_ = 1.5 [49], and the infectious duration was around 3.75 days (i.e. recovery rate *γ* ≈ 0.267) [50]. During the outbreak of H1N1 influenza in Hong Kong, there were more than 36 000 laboratory confirmed cases (i.e. as of September 2010), among which about 290 were identified as severe cases (i.e. the reporting rate of severe disease infections 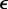 was estimated as 0.805%) [51]. In Hong Kong, the outbreak of H1N1 infection appeared in September 2009 and, in the second wave of infection, there were far fewer infection cases during the winter of 2009–2010. The human swine influenza (HSI) vaccination programme was launched on 1 December 2009. The numbers of vaccinated individuals ever since are shown in figure 3. As of 13 March 2010, more than 180 000 doses of HSI vaccines were administered to persons of various groups [52]. In the whole HSI vaccination programme, a total of 34 cases of AEFI were reported. The rate of AEFI was evaluated as 17.8 per 100 000 vaccinated individuals (i.e. the reporting rate of vaccine-related AEFI *κ* was estimated as 0.0178%) [53].

**Figure 4.**
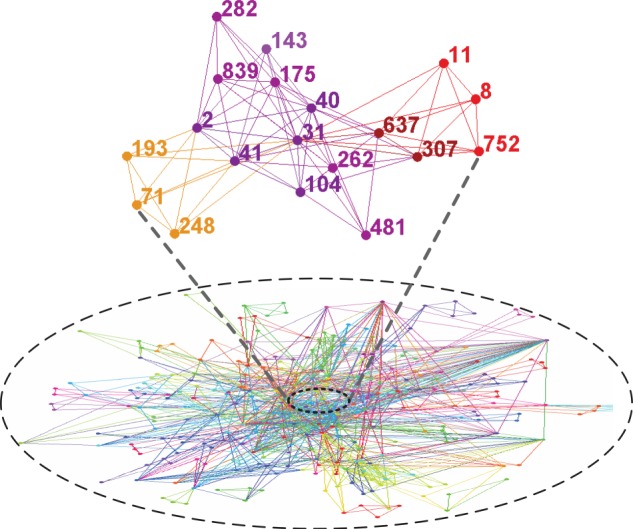
A partial snapshot of individuals' social network. We use a network structure to represent individuals' interaction relationships, based on the data of a Facebook-like online community [54]. In such a network, the nodes denote individuals and the links represent their interactions in terms of sending and receiving messages.

We further construct a social network to characterize individuals' interaction relationships based on the data of a Facebook-like online community [54], in which the registered users can communicate with each other online through personal blogs and forum postings. In this network, there are in total 1899 nodes and 13 838 undirected links among them. As shown in the partial network snapshot of figure 4, the nodes denote the registered users, and the links among them represent their interaction relationships in terms of sending and receiving at least one message. Based on such a network structure, we carry out a series of Monte Carlo simulations and experimentally examine the above-mentioned belief-based characterization of individuals' vaccination decision-making. In our simulation, we assume that the spreads of awareness and disease are simultaneous. Moreover, individuals who have decided either for or against vaccination will no longer change their decisions. Meanwhile, those with the state of ‘yet to decide’ will revise their decision-making in the following time steps (i.e. days). We run the simulations of each considered scenario for 1000 times to remove the stochastic effects on individuals' vaccination decision-making.

**Figure 5.**
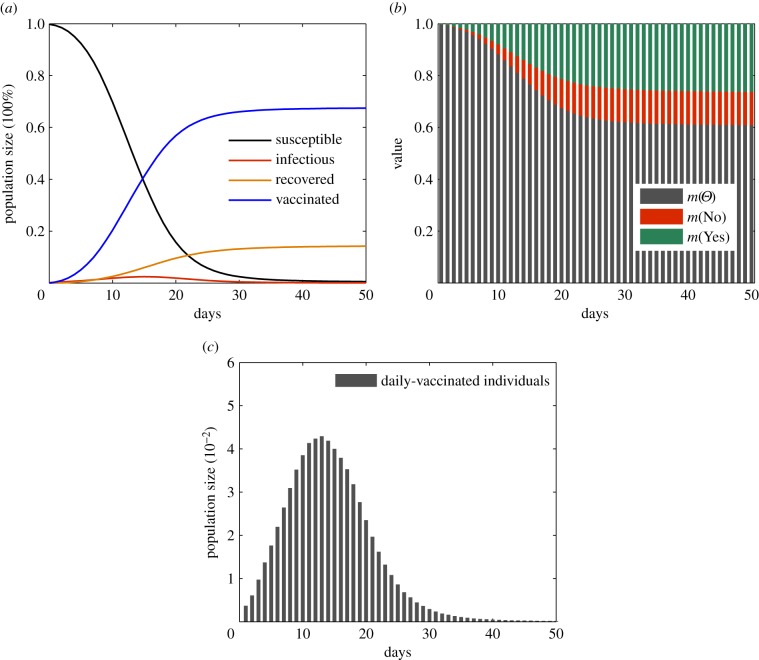
Basic scenarios for the Monte Carlo simulations of disease transmission and voluntary vaccination. (*a*) The dynamics of disease transmissions in terms of the sizes of susceptible, infectious, recovered and vaccinated populations. (*b*) The average belief values about vaccination in a host population, i.e. *m*(Yes), *m*(No) and 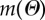. (*c*) The dynamics of voluntary vaccination in terms of the number of daily-vaccinated individuals.

The simulation results in figure 5 show the dynamics of disease transmission and individuals' voluntary vaccination for the first 50 days. In this scenario, we examine the patterns of the vaccination programme in terms of daily-vaccinated individuals. We observe that the number of vaccinated individuals increases steadily in the earlier days of a vaccination programme as individuals' uncertainty about vaccination decreases. However, the reported cases of vaccine-related AEFI significantly increase individuals' belief about non-vaccination, which leads to a sharp decrease in the number of daily-vaccinated individuals after it has peaked on day 12.

### 3.2. The interplay of two dynamics

We run the proposed model with the above-mentioned parametrizations under various scenarios to reveal the interplay between the dynamics of disease transmission and individuals' vaccination. In doing so, we investigate the impacts of the spread of awareness about disease severity and vaccine safety in a host population by means of investigating various settings of the reporting rates of disease- and vaccine-related events 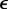 and *κ*, and the fading coefficient of awareness spread *ρ*.

**Figure 6.**
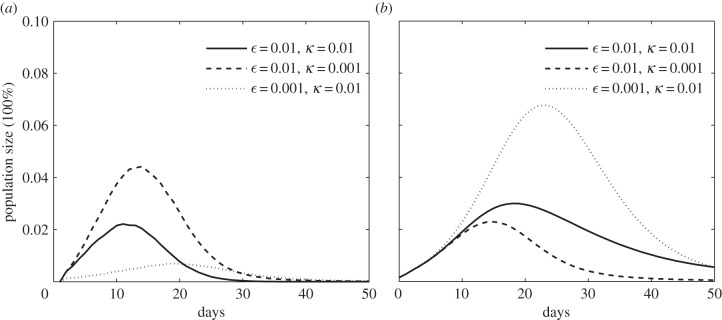
The impacts of reporting rates of disease- and vaccine-related events (i.e. severe disease infections 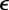 and vaccine-related AEFI *κ*). (*a*) The dynamics of voluntary vaccination (i.e. the number of daily-vaccinated individuals). (*b*) The dynamics of disease transmissions (i.e. the number of infectious individuals on each day).

As shown in figure 6, we first investigate the reporting rates for negative events on severe disease infections (i.e. 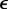) and vaccine-related AEFI (i.e. *κ*) with respect to two levels: 1% and 0.1%. Here, we set 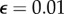 and *κ* = 0.001 for the situation of disease scare, and similarity, 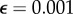 and *κ* = 0.01 for the scenario of vaccine scare. Generally speaking, a relative higher reporting rate of severe disease infections will prompt individuals' tendency for vaccination (i.e. as shown in figure 6*a*, dashed curve), which will in turn reduce disease transmissions (i.e. as shown in figure 6*b*, dashed curve). Moreover, vaccination in the early stage will be more effective than that in the later stages. We can observe that when 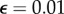, the difference in the number of vaccinated individuals between the situations of *κ* = 0.001 and *κ* = 0.01 (i.e. as shown in figure 6*a*, dashed curve and solid curve, respectively) is relatively small for the early stage of disease transmissions (i.e. before day 10). After that, the vaccination dynamics when 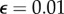 and *κ* = 0.001 will peak at the level of more than 4% of individuals who will vaccinate themselves on day 15, whereas that of 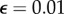 and *κ* = 0.01 will peak at 2% on day 11. Accordingly, we can observe that the disease dynamics in the situations of 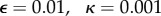 and 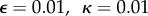 (i.e. as shown in figure 6*b*, dashed curve and solid curve, respectively) have relatively low incidence rates at the peaks of disease infection, whereas the lasting periods of disease transmissions are different.

**Figure 7.**
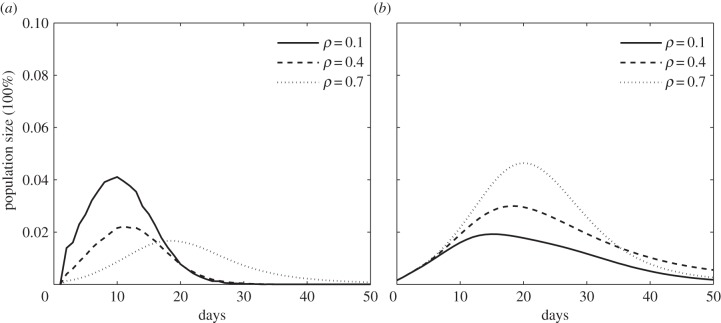
The impacts of fading coefficient (i.e. *ρ*) during the spread of awareness in a host population. (*a*) The dynamics of voluntary vaccination (i.e. the number of daily-vaccinated individuals). (*b*) The dynamics of disease transmissions (i.e. the number of infectious individuals on each day).

Besides, we have investigated the effect of awareness fading with respect to different fading coefficients *ρ*, the results of which are shown in figure 7. We note that awareness fading can affect the dynamics of individuals' vaccination in terms of the number of vaccinated individuals and the time of individuals' vaccine administration. In our simulation, when the fading coefficient *ρ* = 0.1, the number of daily-vaccinated individuals will peak on day 10 with the rate around 4% (i.e. as shown in figure 7*a*, solid curve). The vaccination rates will be around 2% and peak on day 12 and day 19, if the fading coefficients are set as *ρ* = 0.4 and *ρ* = 0.7, respectively (i.e. as shown in figure 7*a*, dashed curve and point curve). In this situation, we can observe that the spread of awareness with a weak fading effect (i.e. a smaller fading coefficient) will prompt individuals' vaccination and thus prevent disease transmissions effectively (i.e. as shown in figure 7*b*, solid curve when *ρ* = 0.1).

## 4. Discussion

It has long been observed that the spread of awareness will affect individuals' health-related behaviour. For instance, individuals who are aware of disease infections may take measures to reduce their susceptibility or distance their social contacts to prevent themselves from disease infections [47,55]. In the context of vaccination, the spread of awareness about severe disease infections and vaccine-related AEFI will affect individuals' perceptions about disease and vaccine and, hence, substantially change their vaccination behaviour.

In understanding the public acceptance of a vaccination programme, empirical studies have identified a series of determinants associated with individuals' vaccination decisions, such as the previous experience of vaccination [56], the perceived risk of disease infection as well as the safety and efficacy of vaccine [10,57–59], the social influence from professional instructions or friends' recommendations [9,56,58] and the socio-economic-related considerations [9,15]. Mathematical models have been developed to describe individuals' vaccination decision-making during the spread of an infectious disease [16,60]. As mentioned earlier, the pay-off-based approaches that use the game-theoretical analysis have characterized individuals' rational vaccination decisions by means of exploring the herd immunity effects (i.e. vaccinating a proportion of the host population would decrease the infection risk for the rest of unvaccinated individuals [5,61]). For instance, Bauch *et al.* [14,17] characterized individuals' voluntary vaccination as a modified minority game in favour of optimizing personal pay-offs. Cojocaru [62] extended the game-theoretical analysis of vaccination to a heterogeneous host population group. Perisic *et al.* [18,63] further examined individuals' vaccination game with respect to their contact network structures. Besides, social and psychological aspects of human behaviour have also been taken into consideration, such as a social learning process [20] and imitation behaviour [21–24].

As a further step from the above-mentioned studies, here we have considered a belief-based characterization of individuals' vaccination decisions. In our proposed model, we have correlated individuals' subjective assessment of disease severity and vaccine safety with the dynamics of disease transmission and voluntary vaccination by exploring the awareness about disease and vaccine. Different from the existing belief-based studies, e.g. that of Coelho *et al.* [25], we have characterized individuals' belief value updates as a result of the spread of awareness in a structured host population (i.e. the social network from an online community). In this case, we can represent the situation that individuals collect health-related information from online social media, and make the vaccination decisions according to their obtained awareness from socially connected neighbours [37,44]. Additionally, instead of the polarized decisions, i.e. either vaccination or not, we have introduced the third decision response in terms of ‘yet to decide’ and associated it with the belief value of uncertainty. By doing so, we have used and extended the DST to characterize individuals’ belief value updates in the presence of uncertainty.

By parametrizing the proposed model with the epidemiological scenario of the 2009 Hong Kong H1N1 influenza epidemic, we have carried out a series of simulations on disease transmissions and voluntary vaccination. Based on that, we have investigated the impacts of the spread of awareness on individuals' vaccination decisions with respect to two considered impact factors. First, the reporting rates of severe disease infections 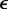 and vaccine-related AEFI *κ* were used to represent the frequencies of respective topics that will draw public attention on social media. Our simulation results have shown that the reporting rates will determine the number of vaccinated individuals. Second, we considered the fading coefficient of awareness spread *ρ*, a parameter used to quantify the effect of certainty decay during the information flows among individuals. We have observed that fading coefficient can affect the time at which individuals will make their decisions as to taking vaccine or not accordingly. Specifically, a higher fading coefficient (i.e. a faster certainty decay) will significantly delay individuals' vaccination decision-making, which will, in turn, influence the coverage of a vaccination programme. Salathe *et al.* [37] have earlier studied vaccination sentiments with online social media (i.e. Twitter) and have found that individuals' behaviour of sharing the same sentiment was correlated with the frequency of information flows among them.

Our study on computationally characterizing the impacts of the spread of awareness has practical implications for public health authorities to predict the extent of public acceptance of a vaccination programme in advance by exploring the decision-making models. In recent years, a growing number of individuals use the Internet-based communication services to obtain and share the health-related information [64,65]. This represents the growing power of analysing individuals' online communication data to track the events in real time during an epidemic. Salathe *et al.* [37] collected individuals' communication messages from Twitter and assessed the public sentiments towards a novel vaccine. Henrich *et al.* [44] used online comments to capture public attitudes about the H1N1 vaccine. Online social media have become an effective means for sharing and creating public perceptions about disease and vaccine, upon which our proposed vaccination decision-making models can readily be used to estimate public acceptance of a vaccination programme [42,66]. Thereafter, public health authorities will be able to adjust their vaccination strategies drawing on the model-based decision-making analysis, so as to improve the effectiveness of adopted strategies.

So far, our study has provided a modelling framework that incorporates the spread of awareness with the belief-based characterization of decision-making. It should be pointed out that the obtained results may depend on the specific assumptions that were made in the design of our model. First, we have assumed no prior knowledge about disease and vaccine. However, individuals' historical experience, e.g. vaccination against seasonal influenza, may affect their vaccination decisions in the face of an emerging infectious disease, e.g. the 2009 H1N1 influenza. We have also assumed that vaccination dynamics and disease dynamics were instantaneously developed as well as individuals' vaccination decision-making was carried out simultaneously. Individuals' asynchronized decision-making and delayed vaccination could affect the simulation results. In our proposed model, the spread of awareness only accounts for individuals' localized interactions (i.e. a Facebook-like online community as used in our example), whereas the global effect of public media has not been taken into account in this study. It would be interesting to extend the current model by incorporating a globalized spread of awareness; that is to say, each individual will be aware of a reported event of disease and vaccine with a certain probability.

Besides the above-mentioned limitations in our decision-making modelling, we have adopted a simplified SIR-based epidemic and vaccination model, in which vaccine efficacy and the possible lag between the vaccine administration and the attainment of immunity were not taken into account. Furthermore, infected individuals in the latency state with no symptoms could make mistakes in their decisions. These issues are worth further investigations in our future work.
